# A structured five-step collaborative inquiry approach to mathematical reasoning in elementary school: effects and implementation evidence

**DOI:** 10.3389/fpsyg.2026.1813634

**Published:** 2026-08-14

**Authors:** Huan Zeng, Jingwen Feng, Meng Dai

**Affiliations:** 1 Faculty of Education, Sichuan Normal University, Chengdu, China; 2 Faculty of Education, Shinawatra University, Pathum Thani, Thailand

**Keywords:** collaborative inquiry, elementary mathematics, mathematical reasoning, quasi-experimental design, teaching intervention

## Abstract

Mathematical reasoning is a core goal of elementary mathematics, yet classroom opportunities to conjecture, justify, and evaluate claims remain uneven. This study examined the effects and implementation of a Structured Five-Step Collaborative Inquiry-Based Instructional Model (CIBI) in Grade 4 mathematics. Using a pretest–posttest nonequivalent control-group design, 96 students (Class A, *n* = 47; Class B, *n* = 49) in a public school in Chengdu, China received either 5 weeks of CIBI (20 lessons) or business-as-usual instruction. Reasoning was assessed with a 10-item open-ended Mathematical Reasoning Test using parallel forms (αA = 0.964, αB = 0.967; form equivalence *r* = 0.993) and analytic scoring (ICC = 0.994). Observation-based fidelity differentiated conditions (*M* = 1.10 vs. 0.48, 0–3). Mixed-effects modeling showed greater pre–post gains under CIBI (time × condition *b* = 3.76, *p* < 0.001), and posttest differences favored CIBI (*d* = 0.66); the adjusted advantage at the mean pretest was 3.84 points. Findings were robust to covarying midterm achievement and indicated improvements across conjecturing, evidence use, justification, and near-transfer. Overall, CIBI offers a practicable scaffold for strengthening elementary reasoning.

## Introduction

Mathematical reasoning is widely positioned as a central goal of school mathematics because it enables learners to go beyond executing procedures toward constructing, connecting, justifying, and evaluating mathematical claims. Consistent with contemporary conceptualisations, mathematical reasoning involves processes such as conjecturing, generalising, comparing, justifying, and proving, as well as the discursive structures that make those processes visible and accountable in classroom talk and writing (Jeannotte and Kieran, 2017; Lithner, 2008; Stylianides, 2007; Xu and Fan, 2024). In elementary grades, reasoning is particularly consequential: early participation in explaining why a method works, coordinating representations, and defending claims can shape students’ mathematical identities and the kinds of learning opportunities they take up later (Jeannotte and Kieran, 2017). Reasoning, therefore, is not an optional extension to content learning but an integral part of meaningful mathematical understanding from the early grades, and it also aligns with broader educational concerns about cultivating students’ twenty-first-century skills (Watcharinrat et al., 2024).

Yet, despite policy-level emphasis, research repeatedly documents that opportunities for students to reason are uneven and often constrained in everyday classrooms. Analyses of elementary instruction show that lessons can be dominated by short-answer exchanges and procedural completion, with limited space for students to develop and warrant claims, interrogate peers’ ideas, or refine arguments in response to critique (Banerjee and Stacey, 2025). This gap suggests that “reasoning” must be actively organised through task design, discourse norms, and evaluative criteria rather than treated as a natural by-product of doing mathematics. In many systems, teachers also report substantial difficulty in operationalising “reasoning” into actionable classroom practices, and observations indicate that reasoning opportunities that arise spontaneously are not consistently leveraged (Makar, 2024; Mukuka et al., 2023). Indeed, specific teacher actions are critical in shaping and sustaining mathematical argumentation in the classroom (Bredow and Knipping, 2025). This makes the central challenge not simply whether reasoning is valued, but how it is instructionally organised in everyday lessons.

One promising direction is collaborative inquiry, in which students investigate mathematically rich problems, generate conjectures, and coordinate evidence through dialogic interaction. A key advantage of collaboration is that it makes thinking public: students are required to externalise strategies, negotiate meanings, and respond to others’ challenges—conditions that are foundational for building justifications and proofs (Conner et al., 2014; Mercer and Sams, 2006). However, inquiry and group work do not automatically produce high-quality reasoning. Without explicit structure, students may remain at the level of sharing answers, divide labour without engaging with each other’s ideas, or treat justification as an afterthought rather than as a central mathematical practice. Research on inquiry-based learning similarly indicates that guidance and scaffolding are critical if students are to move beyond activity participation toward disciplined reasoning (Hmelo-Silver et al., 2007; Lazonder and Harmsen, 2016). Thus, the key issue is not whether inquiry matters, but how inquiry lessons are structured so that exploration leads to explicit claim-building, justification, critique, and consolidation.

This issue becomes clearer when prior approaches to organising inquiry lessons are considered. Inquiry-based mathematics education has long emphasised students’ exploration of meaningful tasks and teachers’ support for collective sense-making, which has proven broadly effective for conceptual understanding (Artigue and Blomhøj, 2013; Mediana et al., 2025). Discussion-based approaches have likewise shown the value of launching rich problems, monitoring students’ work, selecting and sequencing student solutions, and connecting ideas in whole-class discussion (Stein et al., 2008). More broadly, structured lesson frameworks such as the 5E model organise learning through phases including engagement, exploration, explanation, elaboration, and evaluation (Bybee et al., 2006). These traditions have established the importance of exploration, discussion, and consolidation. However, they often operate at a relatively broad level and do not always specify with sufficient precision how teachers can support the movement from initial idea generation to collaborative comparison, explicit justification, critique of alternatives, and public evaluation of claims in elementary mathematics classrooms.

Against this backdrop, the present study proposes and evaluates a Structured Five-Step Collaborative Inquiry-Based Instructional Model (CIBI) designed to strengthen elementary students’ mathematical reasoning. The model preserves the productive core of inquiry (student sense-making, conjecture generation, and evidence-based validation) while adding explicit, repeatable scaffolds for collaboration and argumentation. Conceptually, CIBI treats reasoning as a social and epistemic practice: students co-construct claims, coordinate evidence, and engage in public evaluation of competing arguments. Empirically, the study tests whether implementing CIBI in Grade 4 mathematics improves students’ mathematical reasoning relative to business-as-usual instruction, using a pretest–posttest quasi-experimental design with a comparison group. The contribution of the present model is not to replace inquiry, but to increase its instructional precision by distinguishing stages that are often merged in prior frameworks, especially the movement from initial idea generation to collective justification and public evaluation of claims.

In addition to outcome evaluation, the study responds to calls for more implementation-oriented evidence in reasoning and proof research. A systematic review of proof and proving across school and university contexts underscores the field’s need for studies that connect instructional design, enactment, and learning outcomes, rather than treating classroom practices as a black box (Stylianides et al., 2024). Accordingly, the present work couples impact estimation with implementation evidence (e.g., observation-based indicators of step enactment and argumentation quality, and artefacts that document students’ claims and warrants), allowing interpretation of effects in light of how the model was actually realised.

The study addresses two hypotheses:

*H1*: Controlling for pretest scores, students in the CIBI group will demonstrate significantly higher posttest mathematical reasoning than students in the comparison group (tested via ANCOVA or an equivalent linear mixed model).

*H2*: Students in the CIBI group will show a significant pretest-to-posttest improvement in mathematical reasoning, whereas gains in the comparison group will be smaller or non-significant.

## Literature review

### Mathematical reasoning in elementary school

Mathematical reasoning (MR) is widely understood not as a single skill but as a set of epistemic practices through which learners generate, connect, justify, and evaluate mathematical claims. Research on school mathematics has conceptualised reasoning as including conjecturing, generalising, comparing, justifying, and proving, while also emphasising that these processes become educationally significant when they are made visible in classroom discourse (Jeannotte and Kieran, 2017; Lithner, 2008; Stylianides, 2007). From this perspective, reasoning in elementary school is not limited to formal proof; it also includes explaining why a strategy works, identifying regularities across cases, and evaluating whether a claim is mathematically warranted. Although primary-age students are capable of substantial reasoning, research shows that opportunities to engage in such activity are unevenly distributed across classrooms. Studies of elementary instruction indicate that many lessons are organised around short-answer recitation and procedural completion, leaving limited space for students to compare methods, justify claims, or critique alternatives (Stein et al., 2008; Yackel and Cobb, 1996). Classroom-analytic work further suggests that reasoning opportunities depend not only on the cognitive demand of tasks but also on whether teachers press students to make claims explicit and support them with evidence (Jeannotte et al., 2021; Valenta et al., 2024). In this sense, MR should be treated as an explicit instructional target rather than an assumed by-product of task completion.

Engagement is also important for the development of MR in the elementary grades. Barnes (2021) shows that enjoyment and perseverance are closely tied to students’ willingness to remain involved in demanding reasoning activity. For young learners, support for reasoning is therefore not only cognitive and discursive but also motivational: classrooms must sustain participation long enough for conjectures to be tested, revised, and justified.

### From collaboration to collective argumentation

A substantial body of research suggests that collaboration can support MR when interaction is organised around mathematical explanation rather than mere task completion. Collaborative settings can make students’ thinking public, creating opportunities to compare strategies, request clarification, and challenge claims; these interactions, in turn, can support the co-construction of justification (Conner et al., 2014; Mercer and Sams, 2006; Webb et al., 2014). However, this potential is not automatic. Studies of group work repeatedly show that students may divide labour, exchange answers, or accept ideas uncritically unless discussion is structured to focus on reasoning and evidence (Webb et al., 2014; Hmelo-Silver et al., 2007).

For this reason, productive collaboration has increasingly been conceptualised as collective argumentation rather than simple participation in group work. In collective argumentation, students are expected to propose claims, respond to challenge, refine warrants, and evaluate alternatives in relation to shared mathematical norms (Conner et al., 2014). Importantly, teacher orchestration is central to this process. Research shows that teachers shape what counts as an acceptable argument through the questions they ask, the solutions they select for public discussion, and the extent to which they press students to explain why a claim should be accepted (Mata-Pereira and da Ponte, 2017; Stein et al., 2008; Zhuang and Conner, 2022). Thus, collaboration supports reasoning most effectively when classroom talk is organised around explicit claim-evidence-warrant relations and when critique is normalised as part of doing mathematics.

### Why a structured five-step inquiry model

Inquiry-based pedagogies are often proposed as a way to strengthen MR because they foreground sense-making, conjecture generation, and justification. Yet research also shows that inquiry-based learning does not by itself guarantee high-quality reasoning. Reviews of inquiry-based learning indicate that guidance and scaffolding are critical if students are to move from active participation to disciplined explanation and justification (Hmelo-Silver et al., 2007; Lazonder and Harmsen, 2016; Calleja et al., 2024).

Relatedly, evidence from teachers’ reported and observed practices suggests that valuing reasoning is insufficient without concrete instructional tools. Mukuka et al. (2023) show that teachers may perceive themselves as supporting students’ reasoning, while observations indicate that opportunities to foster and assess reasoning are not consistently used.

These findings help explain why a more structured model is needed, especially in elementary classrooms. Existing inquiry approaches have established the importance of exploration and discussion, but they often remain broad in specifying how students move from initial idea generation to collaborative comparison, explicit justification, critique of alternatives, and public evaluation of claims. A structured model can reduce this gap by embedding reasoning checkpoints into predictable lesson phases and by clarifying what students are expected to produce at each stage. Such structure does not imply scripting student thinking; rather, it supports the teachability and observability of reasoning in everyday classroom practice.

### Positioning the CIBI model in relation to existing inquiry frameworks

The contribution of CIBI is clearer when it is positioned against prior lesson structures. General inquiry-based teaching emphasises student exploration and sense-making, but often leaves the pathway from exploration to justification under-specified (Artigue and Blomhøj, 2013). Exploration-centred or problem-solving lesson structures provide practical flow for launching tasks and discussing solutions, yet they do not always distinguish clearly between generating ideas, collaboratively testing those ideas, and publicly evaluating claims. Discussion-based frameworks such as the five practices are powerful for orchestrating whole-class discourse, but their primary focus is discussion management rather than a stepwise model designed specifically for MR development (Stein et al., 2008). Similarly, broad phase models such as the 5E framework offer a clear inquiry cycle, but categories such as “exploration” or “explanation” may still encompass multiple reasoning processes that need to be separately supported in mathematics classrooms (Bybee et al., 2006).

Against this background, CIBI contributes at three levels. First, it increases instructional granularity by distinguishing phases that many prior frameworks treat more broadly, especially the movement from idea generation to comparison, justification, critique, and evaluation. Second, it is designed explicitly for mathematical reasoning rather than for inquiry in a general sense, making claim construction and validation central rather than incidental. Third, it places collaborative argumentation at the centre of lesson design by structuring peer interaction around claim-evidence-evaluation sequences. The value of the model, therefore, is not that it replaces existing inquiry approaches, but that it makes their reasoning demands more explicit, repeatable, and observable for elementary classrooms.

## The five-step collaborative inquiry-based instructional model (CIBI)

### Design principles

CIBI is a structured, lesson-level model for organising collaborative inquiry so that students repeatedly engage in two core reasoning demands: (a) developing mathematical claims through exploration and comparison, and (b) validating claims through justification, critique, and revision (Jeannotte and Kieran, 2017; Valenta et al., 2024). The model is “structured” in the sense that each phase has an explicit epistemic goal, a predictable student product, and a small set of teacher discourse moves that press for explanation and connection (Ellis et al., 2019). At the same time, the model is inquiry-based because students’ conjectures, representations, and partial explanations are treated as legitimate starting points for collective work rather than as deviations from a predetermined solution path; this is consistent with inquiry traditions that view students’ ideas as resources for mathematical sense-making (Artigue and Blomhøj, 2013; Hmelo-Silver et al., 2007).

CIBI operationalises collaboration as collective argumentation, not mere group work. Across phases, students are positioned to propose claims, request reasons, provide evidence, and evaluate the adequacy of warrants (Conner et al., 2014). Teacher actions are therefore central: teachers frame what counts as an acceptable justification, help students distinguish examples from evidence and evidence from explanation, and manage critique so that it remains mathematical and respectful (Ellis et al., 2019; Mata-Pereira and da Ponte, 2017; Zhuang and Conner, 2022). Thus, CIBI specifies both activity structures and reasoning expectations.

**Figure 1:**
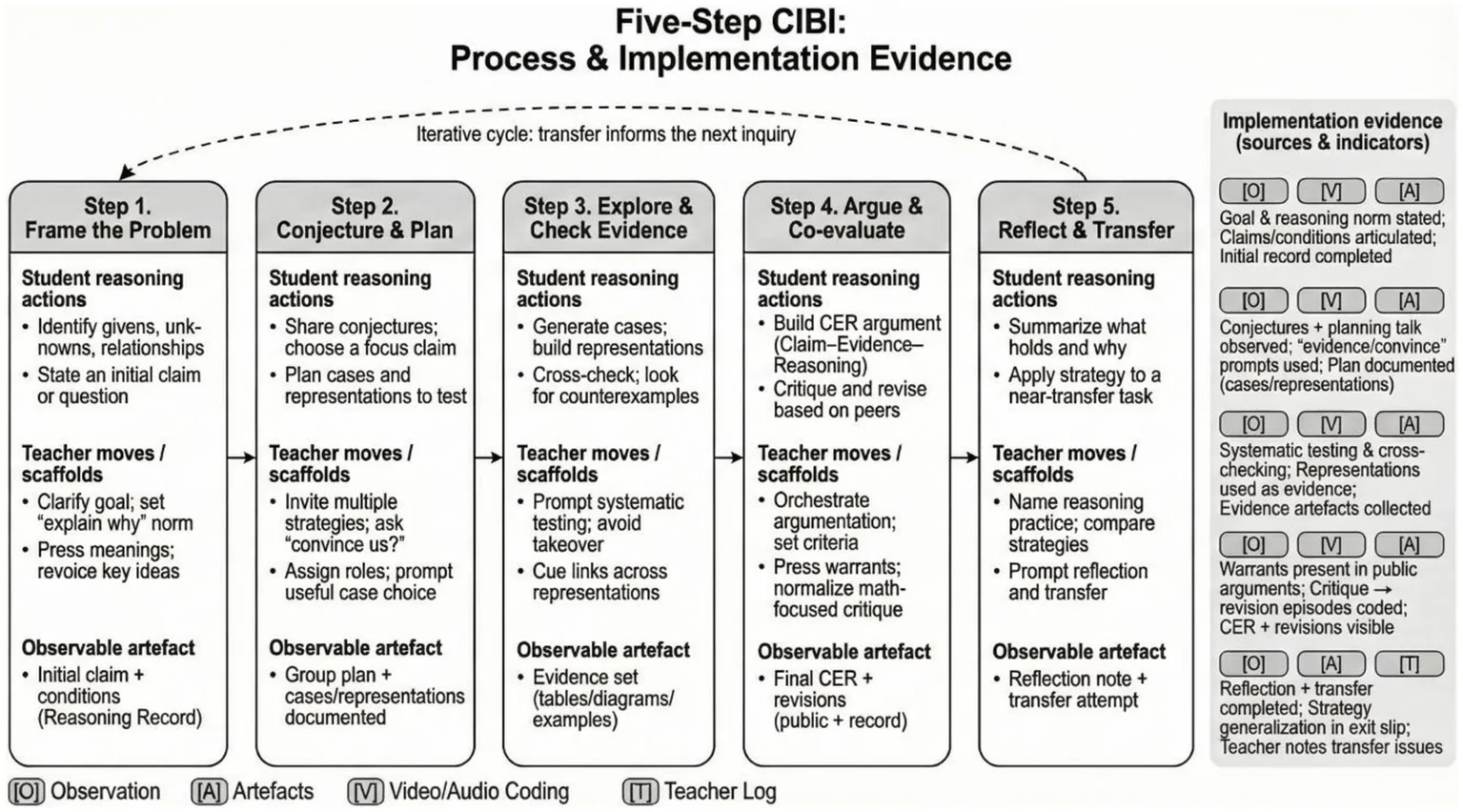
Five-Step CIBI: Process and implementation evidence. The figure summarizes the Structured Five-Step Collaborative Inquiry-Based Instructional Model (CIBI). The left panel depicts the five-step instructional cycle (framing → conjecturing/planning → exploring/checking evidence → arguing/co-evaluating → reflecting/transferring) and its iterative loop. For each step, the figure specifies the intended pedagogical purpose, the reasoning function, the collaborative/discursive function, the teacher scaffolds, and at least one observable artefact.

The Structured Five-Step Collaborative Inquiry-Based Instructional Model (CIBI) operationalizes mathematical reasoning as a set of teachable classroom practices that recur across lessons. Specifically, CIBI sequences students’ epistemic work from problem framing to conjecturing, evidence generation, argumentation, and transfer, while pairing each phase with corresponding teacher scaffolds and observable student products. Figure 1 provides a schematic overview of the model, showing (a) the five-step instructional flow and its iterative cycle and (b) the implementation-evidence mapping used to document enactment (e.g., observation indicators, artefacts, discourse coding, and teacher logs). In the sections below, we first specify the design principles underlying CIBI, then describe each step in terms of student reasoning actions, teacher moves, and expected artefacts, and finally outline how implementation evidence is collected and aligned to the five steps.

### Implementation format

CIBI is intended for regular elementary mathematics lessons and can be used across topics by selecting tasks that allow multiple strategies and invite generalisation. Students work in stable small groups (typically 3–4) with rotating roles such as proposer, challenger, recorder, and connector. Each lesson uses a Reasoning Record that captures (i) initial claim(s), (ii) representations and examples/counterexamples, (iii) warrants linking evidence to claims, and (iv) a refined conclusion and transfer prompt. The Reasoning Record functions as both a cognitive scaffold and an implementation artefact, making visible whether ideas have been produced, examined, justified, and revised.

#### Step 1: context launch and shared problem framing

The lesson begins with a brief launch that establishes a common problem space and clarifies what is being investigated. The teacher introduces a mathematically rich situation and elicits initial interpretations so that the class reaches a shared understanding of key constraints and what would count as a solution or explanation. Rather than immediately soliciting answers, the teacher surfaces candidate claims and uncertainties and signals that the lesson values explanations and justifications, not only correct results. Students individually note an initial conjecture or question on the Reasoning Record.

Step 1 serves a framing function: its pedagogical purpose is to establish a shared object of inquiry, its reasoning function is to make the target claim explicit, and its collaborative function is to prepare students to enter group work with an initial position. Research shows that reasoning opportunities increase when claims, assumptions, and expectations for explanation are made explicit early in the lesson (Valenta et al., 2024). Practical markers include written initial conjectures and teacher prompts such as “What exactly are we trying to decide?”

#### Step 2: conjecture generation and plan co-construction

In groups, students share initial conjectures and select one or two to pursue. They then plan how to test or elaborate the conjecture, deciding what examples, representations, or transformations to generate. The aim is not yet to justify a final conclusion but to make the inquiry process intentional: students explain why a chosen representation might help them see structure. Teachers circulate to press for the reasoning behind choices and broaden participation through questions such as “What makes that example useful?” or “Can someone restate the conjecture?” (Zhuang and Conner, 2022). The group records its planned approach on the Reasoning Record, including at least one check for judging the conjecture.

Step 2 is primarily generative. Its pedagogical purpose is to help students produce candidate ideas, strategies, and representations; its reasoning function is conjecture generation and initial structuring of inquiry; and its collaborative function is to make possible lines of reasoning visible and jointly owned. Although this step falls within what many inquiry frameworks call exploration, its emphasis is on producing candidate strategies or conjectures rather than defending them (Hmelo-Silver et al., 2007; Mercer and Sams, 2006). Practical markers include multiple candidate ideas, a written plan, and teacher prompts focused on choice and participation.

#### Step 3: structured collaborative comparison and claim examination

Groups carry out their plan by generating cases, constructing representations, and looking for invariants or relationships. Students are required to produce visible evidence—worked examples, diagrams with annotations, or systematic tables—rather than only verbal accounts. This externalisation supports collective sense-making and provides material for critique. The teacher’s role is adaptive: when groups stall, the teacher offers prompts that maintain epistemic ownership while pressing students to compare, question, and refine emerging claims in relation to evidence and peer reasoning (Webb et al., 2014).

Although both Steps 2 and 3 may be labelled “exploration,” they serve different epistemic functions. Step 2 is primarily generative, enabling students to produce candidate strategies or conjectures. Step 3 is primarily justificatory and evaluative, requiring students to compare, defend, question, and refine claims in relation to evidence and peer reasoning. Its pedagogical purpose is to move students from planning inquiry to disciplined examination; its reasoning function is to test, elaborate, or constrain initial conjectures; and its collaborative function is to transform group work from answer-sharing into accountable comparison. Without this distinction, students may generate ideas but never justify them, and teachers may not know when to shift from exploration to argumentation. Practical markers include contrasting cases, explicit comparison talk, and revisions to the initial claim.

#### Step 4: argumentation co-construction and conclusion co-evaluation

This step shifts from group-level claim examination to validation. Groups prepare a public argument consisting of a claim, supporting evidence, and a warrant explaining why the evidence justifies the claim. Whole-class discussion is then organised as collective argumentation: multiple groups present, classmates pose challenges, and arguments are revised in response. Teacher actions are decisive in shaping what counts as a legitimate argument—for example, whether “many examples” is treated as sufficient, whether counterexamples are actively sought, and whether generalisations are tied to structural explanations (Conner et al., 2014; Mata-Pereira and da Ponte, 2017). At this stage, claims are no longer accountable only to the small group but to the classroom community and its mathematical norms.

Step 4 is primarily public, justificatory, and evaluative. Its pedagogical purpose is to turn evidence into argument; its reasoning function is validation; and its collaborative function is peer critique and co-evaluation. Unlike Step 3, where claims are examined within the group, Step 4 requires those claims to be defended, questioned, and revised in a public forum. Research on collective argumentation shows that reasoning becomes especially consequential when ideas are made public and held accountable to mathematical norms (Conner et al., 2014; Jeannotte et al., 2021). Practical markers include explicit claim-evidence-warrant statements, peer challenges, and visible revisions in response to critique.

#### Step 5: collaborative reflection and strategy transfer

The final step consolidates learning and supports transfer. Students reflect on (a) what claim was established, (b) what made the argument convincing, and (c) what reasoning strategy or representation they would reuse in a new problem. Groups complete a short transfer prompt that changes surface features while preserving the underlying structure. This phase is designed to sustain perseverance and positive engagement with reasoning by treating revision and critique as normal, productive parts of doing mathematics (Barnes, 2021). The teacher closes by explicitly naming the reasoning practices enacted (e.g., “We compared cases to test a conjecture,” “We used a diagram to justify a general claim”), reinforcing that MR is a learnable set of practices. This naming function also helps stabilise reasoning norms across lessons by making strategies for conjecturing, evidencing, justifying, and evaluating available for future reuse.

Step 5 serves consolidation and transfer. Its pedagogical purpose is to help students abstract from the immediate task; its reasoning function is meta-reasoning about what counted as convincing and why; and its collaborative function is to stabilise shared norms and reusable strategies. This step is supported by research suggesting that explicit reflection and positive engagement are important for sustaining participation in mathematical reasoning over time (Lowrie et al., 2018). Practical markers include written reflections, transfer responses, and teacher summaries that explicitly name the reasoning practices enacted.

### Linking design, enactment, and evidence

Because reasoning outcomes depend on enactment quality, CIBI is paired with implementation evidence that documents what students actually experienced. In line with calls to connect instructional practice to outcomes in proof and reasoning research and to rigorously assess inquiry-based tasks (Stylianides et al., 2024; Vo and Simmie, 2025), the study can operationalise enactment through: (a) observation rubrics aligned to the five steps (e.g., presence of explicit claims, quality of warrants, frequency of peer critique), (b) artefact analysis of Reasoning Records to trace claim development, and (c) brief teacher reflection logs on which discourse moves were used and why. Such evidence also addresses the common gap between teachers’ intentions and observed reasoning practices noted by Mukuka et al. (2023). Linking each step to observable markers is important methodologically as well as pedagogically: it allows outcome claims to be interpreted in relation to how the model was actually enacted rather than treating implementation as a black box.

## Materials and methods

### Research design

A pretest–posttest nonequivalent control-group quasi-experimental design was used to estimate the effects of the Structured Five-Step Collaborative Inquiry-Based Instructional Model (CIBI) on Grade 4 students’ mathematical reasoning. Random assignment was not feasible because class rosters were administratively fixed; therefore, two intact classes were assigned to experimental (CIBI) or comparison (business-as-usual) conditions. To reduce confounding, both classes were drawn from the same grade cohort in the same school, followed the same instructional timetable and curricular scope during the study window, and were screened to be comparable in baseline achievement using the school midterm mathematics examination. In the main analyses, pretest reasoning scores were controlled statistically.

### Participants and setting

The study was conducted in a public elementary school in Chengdu, Sichuan Province, China. From four Grade 4 intact classes, two were selected to be similar in midterm mathematics performance. The experimental group (Class A) included 47 students (25 boys, 22 girls) and the comparison group (Class B) included 49 students (26 boys, 23 girls), yielding 96 students aged 9–10 years. School records indicated no diagnosed learning disabilities or special educational needs for participating students.

Two in-service mathematics teachers implemented instruction. Both held senior elementary mathematics teaching credentials, had 8 years of Grade 4 teaching experience, and regularly participated in school- and district-level mathematics teaching research activities. The school mathematics panel evaluated the two teachers as broadly comparable in instructional expertise. Each teacher taught only one class (one teacher per condition) to avoid contamination across conditions; meanwhile, weekly pacing and content scope were coordinated to minimize differential content exposure.

Prior to the main study, the mathematical reasoning test was piloted with a third Grade 4 class (Class C; n = 45) to establish parallel-forms reliability and examine the equivalence of Form A and Form B. This class was conveniently selected from the same cohort in the participating school to ensure that the pilot sample matched Classes A and B in demographic profiles and baseline achievement, though they did not participate in the actual intervention.

### Procedure and intervention

The intervention lasted 5 weeks with four 40-min mathematics lessons per week (20 lessons total) delivered within the regular timetable. Pretesting occurred 1 week before the intervention and posttesting occurred within 1 week after completion. All tests were proctored by researchers not involved in instructional delivery.

In the experimental condition, CIBI was used in every intervention lesson. Students worked in stable heterogeneous groups of 3–4 and used a brief Reasoning Record to document claim(s), evidence, warrants, revisions, and a short transfer response. In the comparison condition, the class received regular teacher-directed instruction, including explanation of procedures, guided practice, and homework review. Brief peer talk could occur, but lessons did not follow the CIBI five-step cycle and did not use the Reasoning Record or explicit claim-evidence-reasoning routines.

### Teacher training and classroom enactment

Before the intervention, the experimental teacher attended two face-to-face workshops led by the research team (about 3 h each). Session 1 introduced the Purpose for mathematical reasoning and inquiry-based teaching, the epistemic purpose of each CIBI step, and the use of the Reasoning Record. Session 2 focused on enactment: analysing a sample lesson video, rehearsing lesson planning with a common template, and discussing how to support group talk without taking over students’ reasoning. Materials included a training handout, annotated sample tasks, a model Reasoning Record, and one classroom video excerpt. The teacher received written feedback on one trial lesson plan before the intervention began.

To clarify enactment, Table 1 presents typical teacher prompts used across the five steps: Step 1, “What is the problem asking us to decide?”; Step 2, “What example or representation could help your group test that idea?”; Step 3, “Do these cases support the same claim, or do they suggest a difference?”; Step 4, “What claim can your group defend, and what evidence supports it?”; and Step 5, “What have we learned here that could help with a new problem?” These prompts were intended to orient sense-making, generate strategies, compare evidence, support justification, and promote transfer.

**Table 1:** Descriptive statistics and baseline comparisons.

Variable	CIBI Mean (SD)	Control Mean (SD)	Welch t	*p*	Cohen’s d
Midterm mathematics achievement	81.96 (5.38)	77.65 (5.03)	4.04	<0.001	0.83
MRT pretest total (Form A)	25.91 (7.35)	25.39 (7.45)	0.35	0.728	0.07
MRT posttest total (Form B)	31.04 (5.69)	26.76 (7.22)	3.24	0.002	0.66
MRT gain (post−pre)	5.13 (2.34)	1.37 (0.86)	10.38	<0.001	2.15

### Fidelity of implementation

Fidelity was documented using structured classroom observation. Two trained observers used a CIBI Fidelity Observation Rubric aligned to the five steps (Appendix A). The rubric includes 10 indicators (two per step) rated 0–3 (0 = not observed; 3 = strong enactment) and captures both adherence (whether step practices occurred) and quality (e.g., explicit claims, systematic evidence checking, warrants, and meaningful peer critique). Consistent with recommendations for classroom observation data, inter-rater reliability was estimated using intraclass correlation coefficients (Tong et al., 2020).

Observation sampling followed a pragmatic plan: each week, one experimental lesson and one comparison lesson were observed (5 observations per class; 10 total). To establish inter-rater reliability without extensive double-coding, six of the observed lessons (60%, 6/10) were independently rated by both observers. Agreement on lesson-level fidelity (mean of the 10 indicators) was excellent (ICC(2,k) = 0.996). After each observation, the experimental teacher received brief feedback focused on whether all five steps were enacted and whether student reasoning was made explicit.

## Measures

### Mathematical reasoning test (MRT)

Students’ mathematical reasoning was assessed with a researcher-developed open-ended test comprising two parallel forms (Form A and Form B; Appendices B–D). The test was guided by the view that elementary reasoning includes generating ideas (e.g., conjecturing) and validating them (e.g., justification, refutation, and generalization) (Jeannotte and Kieran, 2017; Valenta et al., 2024). To emphasize reasoning over procedural fluency, all items required written explanations and encouraged use of representations and cases.

Each form contains 10 constructed-response items (40 min; total score = 40) distributed across four reasoning dimensions: (D1) claim/conjecture formulation with conditions, (D2) evidence generation and representation use, (D3) justification and evaluation (including counterexample and argument critique), and (D4) generalization/near transfer (Appendix B). Form A was administered as the pretest and Form B as the posttest for both groups.

### Parallel-form equivalence (class C)

To support form equivalence, Class C completed both forms in a counterbalanced, cross-week design with a one-week interval and no feedback between administrations (Appendix E): NO.1–23 completed Form A in Week 1 and Form B in Week 2, whereas NO.24–45 completed Form B in Week 1 and Form A in Week 2. The two forms showed nearly identical mean performance (mean difference [A − B] = −0.04 points) and a very strong association (r = 0.993). Internal consistency was high for both forms (*α*A = 0.964, αB = 0.967).

### Scoring

Each item was scored 0–4 using an analytic rubric (Appendix D) that distinguishes mathematical validity from reasoning quality and warranting. Two trained raters blind to condition and time point double-scored 25% of scripts (48 of 192 scripts: 24 pretest and 24 posttest) to estimate inter-rater reliability. Inter-rater reliability for the MRT total score was excellent (ICC(2,k) = 0.994). Discrepant scores were resolved through discussion, and remaining scripts were single-scored with periodic cross-checks to prevent rater drift.

### Data analysis

Analyses will be conducted in R (or SPSS) with *α* = 0.05 (two-tailed). Baseline equivalence will be examined using descriptive statistics and group comparisons on pretest MRT scores and midterm mathematics achievement. For H1, the primary impact estimate will be obtained using ANCOVA, with posttest MRT score as the outcome, condition as the factor, and pretest MRT score as the covariate; the homogeneity-of-slopes assumption will be checked via a condition-by-pretest interaction.

For H2, a repeated-measures linear mixed-effects model will be fit with fixed effects for time (pre vs. post), condition, and their interaction, and a random intercept for student. The time-by-condition interaction provides the estimate of differential gains. Effect sizes will be reported as adjusted mean differences and standardized mean differences (Hedges’ g), with 95% confidence intervals.

## Results

### Preliminary checks and measurement evidence

All students who were present at pretesting completed the posttest, and the dataset contained no missing values for midterm achievement, MRT pretest total (Form A), MRT posttest total (Form B), or the classroom observation rubric. Table 1 summarizes descriptive statistics. Baseline reasoning was comparable across groups: the CIBI class and the control class did not differ on pretest MRT totals, *t*(93.9) = 0.35, *p* = 0.728, *d* = 0.07. However, the CIBI class performed higher on the school midterm mathematics examination, *t*(92.9) = 4.04, *p* < 0.001, *d* = 0.83. Given this imbalance, treatment effects were examined using models that adjust for pretest reasoning (primary analyses) and were further subjected to a sensitivity analysis additionally adjusting for midterm achievement (see below).

### Measurement evidence for the mathematical reasoning test

Evidence supporting the Mathematical Reasoning Test (MRT) scoring and form equivalence is reported in Table 2. In the counterbalanced pilot with Class C (*n* = 45), Form A and Form B totals were nearly identical (M_A = 26.82, SD_A = 7.85; M_B = 26.87, SD_B = 8.05). The paired mean difference (Form A − Form B) was small and statistically non-significant [*Δ* = −0.04, 95% CI (−0.34, 0.25), *t*(44) = −0.31, *p* = 0.761], and the two forms were strongly associated (*r* = 0.993). The difference scores did not vary meaningfully by administration sequence (AB vs. BA), *t* = 0.01, *p* = 0.995, suggesting minimal order effects under the one-week interval and no-feedback testing protocol.

**Table 2:** Measurement and reliability evidence.

Metric	Value
Parallel-form equivalence (Class C, *n* = 45): Form A total	*M* = 26.82, SD = 7.85
Parallel-form equivalence (Class C, *n* = 45): Form B total	*M* = 26.87, SD = 8.05
Paired difference (Form A − Form B)	Δ = −0.04, 95% CI [−0.34, 0.25], *t*(44) = −0.31, *p* = 0.761
Form A–B association	*r* = 0.993
Order effect on A–B difference (AB vs. BA)	*t* = 0.01, *p* = 0.995
Internal consistency (Class C)	αA = 0.964; αB = 0.967
MRT inter-rater reliability (48 double-scored scripts)	ICC(2,k) = 0.994
Observation inter-rater reliability (double-rated lessons)	ICC(2,k) = 0.996

Internal consistency was high for both forms (α_A = 0.964; α_B = 0.967). Inter-rater reliability for MRT total scores was excellent across 48 double-scored scripts (ICC(2,k) = 0.994), indicating that the analytic rubric yielded stable scoring across independent raters.

### Implementation fidelity

**Figure 2:**
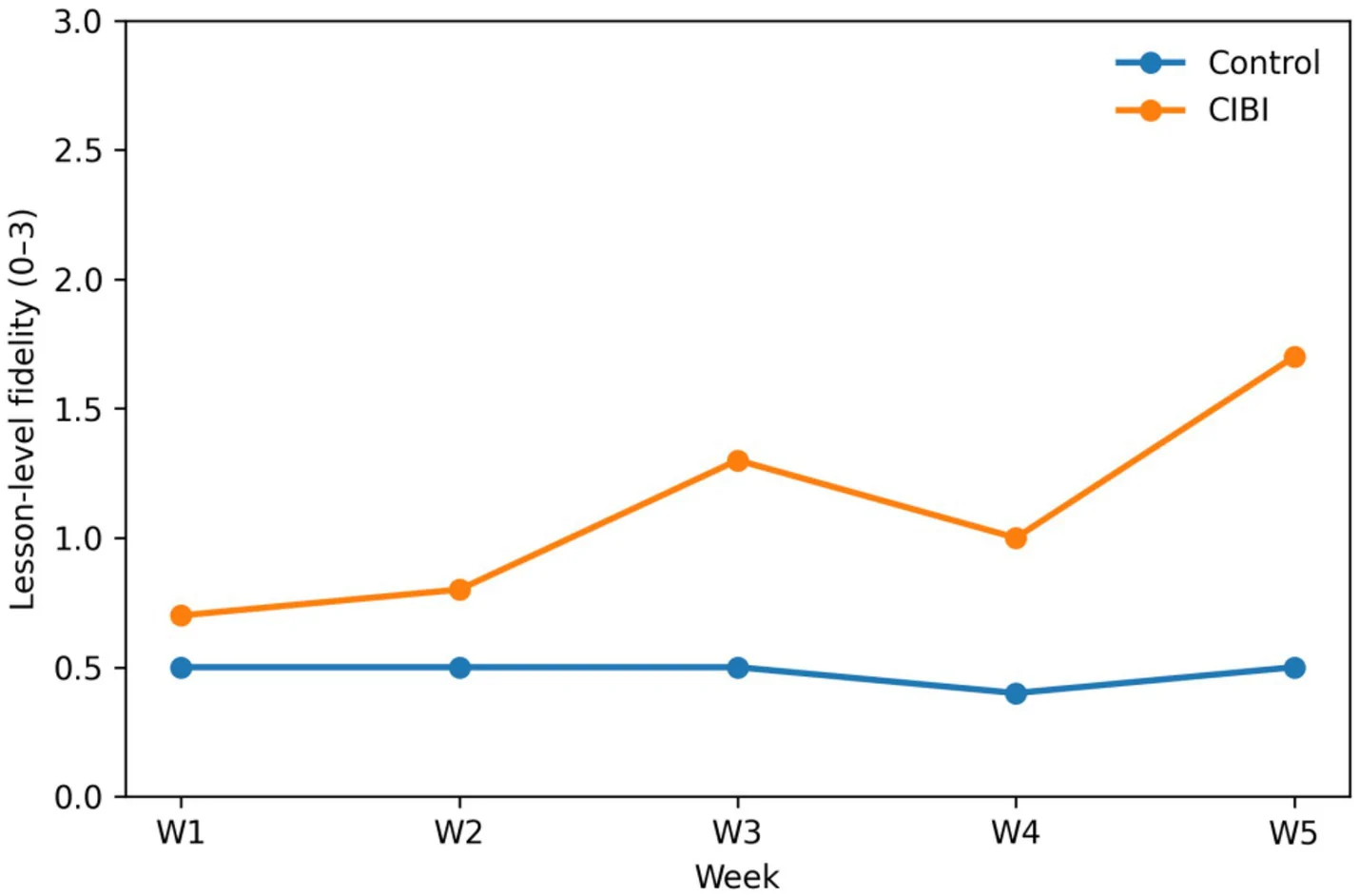
Lesson-level fidelity across 5 weeks (one observed lesson per group per week).

Implementation evidence is summarized in Table 3 and visualized in Figure 2. Across the five observed experimental lessons, overall lesson-level fidelity (mean of 10 indicators, 0–3) averaged 1.10 (0.41), whereas the comparison class averaged 0.48 (0.04). Week-by-week trajectories suggested a gradual strengthening of the CIBI routines, with the experimental class showing increasing scores over time (Figure 2). In contrast, the comparison class exhibited low and relatively stable scores, reflecting that sporadic peer discussion or explanation could occur but the five-step cycle was not enacted systematically.

**Table 3:** Implementation fidelity summary (overall and step-level).

Step/metric	Overall lesson fidelity (mean of 10 indicators)	Frame	Conjecture plan	Explore check	Argue coevaluate	Reflect transfer
CIBI	1.10 (0.41)	1.6	1.1	1.2	0.8	0.8
Control	0.48 (0.04)	1.0	0.0	0.5	0.4	0.5

Step-level profiles (Table 3) indicated that the largest condition differences emerged in the middle phases of the cycle—Conjecture and Plan and Explore and Check Evidence—where CIBI lessons more consistently pressed students to articulate claims, generate and interrogate evidence, and coordinate representations before concluding. These implementation patterns provide convergent evidence that the experimental class received substantively different instructional experiences aligned with the intended CIBI design.

### Effects on mathematical reasoning (MRT total)

**Figure 3:**
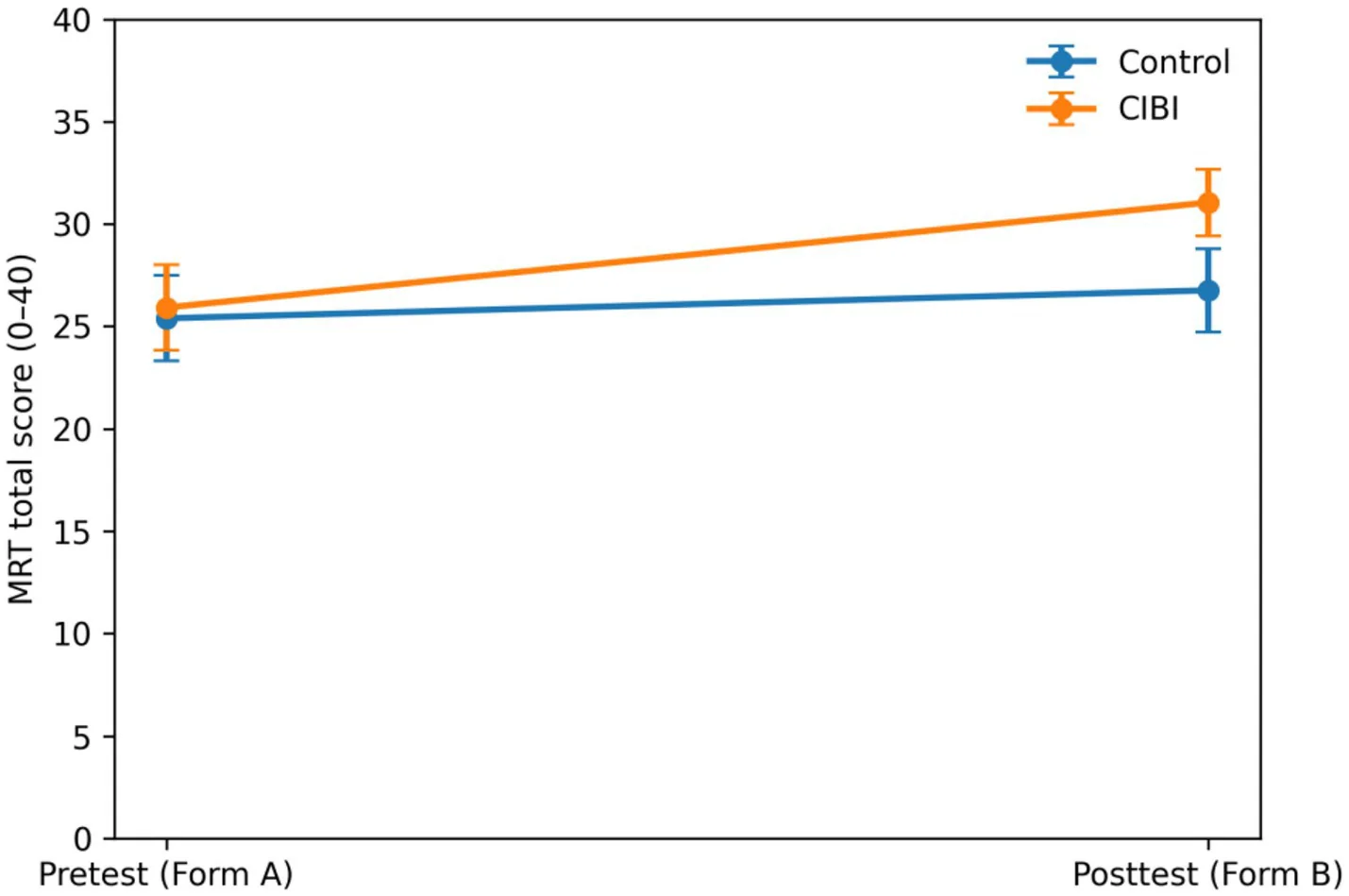
MRT total scores by group and time (95% CIs).

Figure 3 depicts MRT total scores at pretest and posttest. The posttest difference favored the CIBI group: *t*(90.6) = 3.24, *p* = 0.002, *d* = 0.66 (Table 1). More importantly for the pretest–posttest design, the CIBI class demonstrated larger gains from pretest to posttest than the control class (Table 1), *t*(57.8) = 10.38, *p* < 0.001, Hedges’ *g* = 2.14. To reconcile these different effect size magnitudes, although the posttest standardized mean difference indicates a moderate-to-large effect (*d* = 0.66), the effect size based on pre-post gain scores presents a substantially larger magnitude (*g* = 2.14). This divergence is mathematically expected given the pretest baseline: the CIBI group demonstrated an accelerated growth trajectory relative to their initial standing. From an educational perspective, the larger gain-score effect underscores the model’s efficacy in rapidly closing the reasoning gap within a short 5-week intervention, even if the absolute posttest divergence remains within typical ranges for short-term educational interventions.

H2 (differential gains) was tested using a repeated-measures linear mixed-effects model with a random intercept for student. The time × condition interaction was statistically significant (Table 4), *b* = 3.760, SE = 0.353, 95% CI [3.069, 4.452], *p* < 0.001, indicating greater pre–post improvement under CIBI.

**Table 4:** Mixed-effects model fixed effects (MRT total).

Fixed effect	Estimate	SE	*z*	*p*	95% CI
Intercept	25.388	0.986	25.76	<0.001	[23.456, 27.320]
Time[T.post]	1.367	0.247	5.54	<0.001	[0.884, 1.851]
Group[T.CIBI]	0.527	1.409	0.37	0.708	[−2.234, 3.288]
Time[T.post]:group[T.CIBI]	3.760	0.353	10.66	<0.001	[3.069, 4.452]

H1 (posttest differences controlling for pretest) was examined with an ANCOVA-style regression model. The condition × pretest interaction was significant, *F*(1, 92) = 42.91, *p* < 0.001, indicating that the posttest gap depended on baseline reasoning. Accordingly, conditional posttest differences were estimated at representative pretest levels (Table 5). At the sample mean pretest score (25.65), the adjusted posttest advantage for CIBI was 3.84 points. The estimated advantage was larger among students with lower baseline reasoning (5.41 points at M − 1 SD) and smaller but still robust among students with higher baseline reasoning (2.26 points at M + 1 SD).

**Table 5:** Conditional posttest differences (CIBI − Control) at representative pretest levels.

Pretest level	Pretest score	Estimated posttest Δ (CIBI − Control)	95% CI	*p*
Low (M − 1 SD)	18.28	5.41	[4.74, 6.09]	<0.001
Average (M)	25.65	3.84	[3.36, 4.31]	<0.001
High (M + 1 SD)	33.01	2.26	[1.59, 2.94]	<0.001

### Sensitivity analysis controlling for midterm achievement

Because the experimental class scored higher on midterm mathematics achievement at baseline, a sensitivity analysis added midterm achievement as a covariate to the primary models. In the posttest regression model (posttest ~ condition × pretest + midterm), the condition × pretest interaction remained significant, *F*(1, 91) = 42.46, *p* < 0.001. Midterm achievement was also a significant predictor of posttest performance, *F*(1, 91) = 0.02, *p* = 0.881, as expected. Crucially, the estimated CIBI advantage persisted across pretest levels (Table 6): at the sample mean pretest score, the adjusted posttest difference was 3.85 points, with larger estimated benefits for students with lower baseline reasoning (5.43) and smaller benefits for those with higher baseline reasoning (2.28).

**Table 6:** Sensitivity analysis: conditional posttest differences controlling for pretest and midterm achievement.

Pretest level	Estimated posttest Δ (CIBI−Control)	95% CI	*p*
Low (M − 1 SD)	5.43	[4.72, 6.13]	<0.001
Average (M)	3.85	[3.34, 4.37]	<0.001
High (M + 1 SD)	2.28	[1.57, 2.98]	<0.001

In the repeated-measures mixed-effects model including midterm as a covariate (score ~ time × condition + midterm), the time × condition interaction remained significant, *b* = 3.760, SE = 0.353, 95% CI [3.069, 4.452], *p* < 0.001. Together, these results indicate that the CIBI-related differential gains were not explained solely by the baseline difference in general mathematics achievement.

### Supplementary analyses by reasoning dimension (D1–D4)

**Figure 4:**
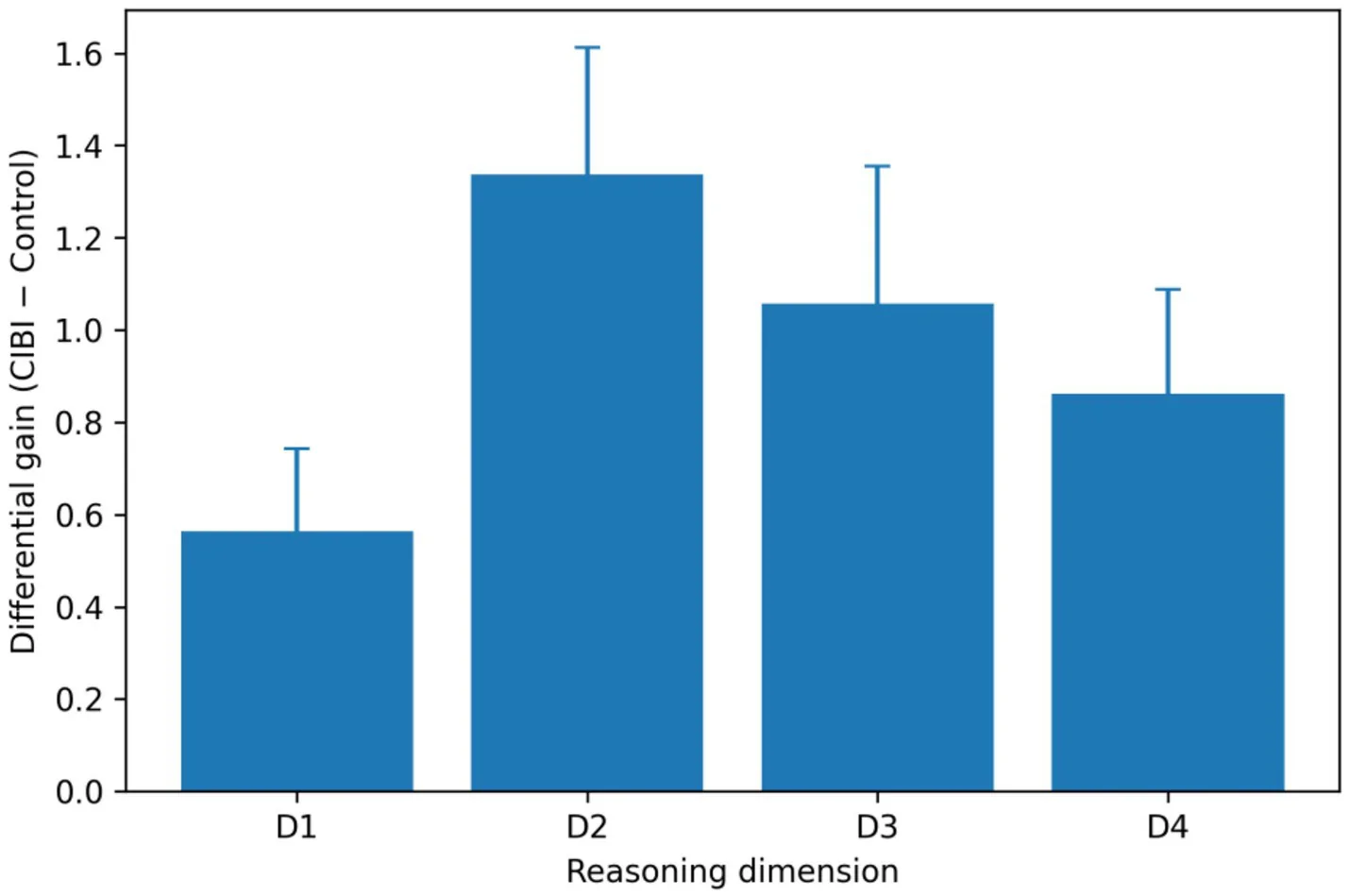
Differential gains by reasoning dimension (CIBI − control) with 95% CIs.

Table 7 provides dimension-level descriptive statistics and differential gains, with Figure 4 summarizing the magnitude of differential gains and 95% CIs. Differential gains favored CIBI across all four reasoning dimensions (all ps < 0.001), indicating broad improvements in conjecturing, evidence use, justification, and generalization/transfer rather than isolated gains in a single component.

**Table 7:** Dimension-level descriptive statistics and differential gains (CIBI − Control).

Dimension (max)	Control Pre M (SD)	Control Post M (SD)	CIBI Pre M (SD)	CIBI Post M (SD)	Δgain (CIBI−Control)	95% CI	*p*	Hedges g (Δgain)
D1 (Calleja et al., 2024)	5.22 (1.67)	5.47 (1.60)	5.30 (1.68)	6.11 (1.55)	0.56	[0.38, 0.74]	<0.001	1.27
D2 (Ellis et al., 2019)	7.63 (2.38)	8.06 (2.31)	7.72 (2.32)	9.49 (1.88)	1.34	[1.06, 1.61]	<0.001	1.97
D3 (Ellis et al., 2019)	7.14 (2.27)	7.47 (2.16)	7.45 (2.26)	8.83 (1.70)	1.06	[0.76, 1.36]	<0.001	1.44
D4 (Calleja et al., 2024)	5.06 (1.63)	5.33 (1.45)	5.19 (1.61)	6.32 (1.29)	0.86	[0.64, 1.09]	<0.001	1.55

The largest differential gains were observed for D2 (Ellis et al., 2019) [Δgain = 1.34, 95% CI (1.06, 1.61)] and D3 (Ellis et al., 2019) [Δgain = 1.06, 95% CI (0.76, 1.36)]. This pattern is consistent with the design of CIBI, which repeatedly requires students to generate evidence (often through multiple representations), evaluate warrants through peer critique, and articulate near-transfer generalizations during the Reflect and Transfer phase.

## Discussion

### Principal findings

This study tested whether a Structured Five-Step Collaborative Inquiry-Based Instructional Model (CIBI) can strengthen Grade 4 students’ mathematical reasoning and whether implementation evidence supports a mechanism-based interpretation. Three findings are central. First, CIBI produced higher posttest reasoning than business-as-usual, with a moderate-to-large between-group difference (d = 0.66; Table 1) and a significant time × condition interaction indicating larger pre–post gains (Table 4; Figure 3). Second, effects were baseline-dependent: adjusted posttest advantages were largest for students with lower pretest reasoning and remained meaningful for higher-baseline students (Table 5). Third, observation-based fidelity data showed that the experimental class enacted substantially more step-aligned practices than the comparison class, with a gradual strengthening of routines across weeks (Table 3; Figure 2).

### Why a structured collaborative inquiry cycle may promote reasoning

Recent work argues that mathematical reasoning does not arise as a by-product of doing problems; rather, classrooms must construct reasoning as a pedagogical object by clarifying what counts as a claim, what counts as evidence, and what warrants are required for acceptance (Alderton and Pratt, 2025; Valenta et al., 2024). CIBI operationalizes these epistemic demands as a repeatable lesson routine. By cycling through framing, conjecturing/planning, evidence checking, public argumentation, and reflection/transfer, the model makes reasoning observable and accountable, aligning with conceptualizations of elementary reasoning as coordinated “searching” and “validating” processes (Jeannotte and Kieran, 2017) that are realized through students’ talk and writing (Jeannotte et al., 2021). Consistent with prior inquiry-based learning, these findings suggest that structure is not the opposite of inquiry-based teaching; rather, structure may preserve inquiry while clarifying what kinds of reasoning work are expected at different moments of the lesson.

The dimension-level results strengthen this interpretation. Differential gains were largest for evidence/representation use (D2) and justification/evaluation (D3; Table 7; Figure 4), which map directly onto the “Explore and Check Evidence” and “Argue and Co-evaluate” phases. This pattern is notable because typical elementary lessons often privilege answer production over sustained evidence-based validation (Banerjee and Stacey, 2025). CIBI likely countered this tendency by embedding teacher prompts that press for explanation and connect representations (Ellis et al., 2019), and by positioning critique as an expected part of group work rather than a social risk. In this sense, the five-step cycle functions as an interactional script that increases opportunities for collective argumentation—claims proposed, challenged, defended, and revised—which is a plausible pathway to stronger warrants and more rigorous evaluation (Conner et al., 2014; Conner, 2022). Educational-psychological research similarly suggests that collaboration benefits learning most when groups are supported to regulate their joint activity through planning, monitoring, and reflection prompts (Michalsky and Cohen, 2021), features that are built into CIBI’s planning and reflection phases. Unlike broader exploration models, CIBI does not leave reasoning expectations implicit; instead, it repeatedly requires students to generate evidence, compare cases, and justify claims in ways that make evaluative reasoning central rather than optional.

Baseline-dependent effects further suggest that structuring may matter most when reasoning demands are otherwise prohibitive. Mathematical reasoning draws on both content knowledge and general inferential processes (Datsogianni et al., 2020). For lower-baseline students, explicit phase goals and shared products (claim–evidence–warrant) may reduce ambiguity about “what to do next” and distribute cognitive work across peers, thereby increasing access to validating practices that are frequently scarce for struggling students (Jeannotte and Kieran, 2017). The conditional effects therefore point to a potential equity mechanism: routinized reasoning norms may broaden participation in high-level practices rather than amplifying initial differences. This finding also suggests that elementary students may benefit from a temporal organization of reasoning actions, in which conjecturing, comparing, justifying, and reflecting are distributed across recognizable lesson phases.

### Implementation evidence and plausibility of mechanisms

The fidelity data add important differentiation evidence: the experimental class experienced systematically different reasoning opportunities aligned with the intended design, while the comparison class remained low and stable. Step profiles indicated the clearest contrasts in planning and evidence checking—phases where unstructured group work often collapses into task completion (Fry et al., 2025). The upward fidelity trajectory also matters conceptually: it implies that CIBI routines were being appropriated and stabilized over time, suggesting that observed learning gains occurred during a period when enactment was still developing. Methodologically, the rubric-based approach with partial double scoring is consistent with guidance for using observations as fidelity indicators in field-based intervention research (Tong et al., 2020), supporting confidence that condition differences reflect systematic instructional contrasts rather than rater idiosyncrasy. These implementation results also suggest that teacher prompts were part of the intervention logic, not merely delivery details, because the quality of reasoning depended on how students were pressed to explain, compare, and justify.

Consequently, while it is difficult to entirely eliminate the influence of individual teacher styles in quasi-experimental designs, the observation-based fidelity indicators (*M* = 1.10 for CIBI vs. 0.48 for control) strongly support our findings. These implementation metrics suggest that the observed group differences (time × condition *b* = 3.76) were primarily driven by the structured five-step approach prescribed by the CIBI model—specifically, the scaffolded movement from idea generation to collective justification—rather than merely by differences in teacher enthusiasm or generic instructional quality.

### Robustness and alternative explanations

Because classes were intact, selection threats remain a legitimate concern. Although the groups were comparable on pretest reasoning, the experimental class scored higher on the school midterm mathematics exam. Sensitivity analyses that additionally adjusted for midterm achievement produced essentially unchanged conditional posttest differences and an unchanged time × condition estimate (Table 6). Midterm achievement also did not explain meaningful additional variance once pretest reasoning was included, indicating that the proximal baseline construct (reasoning) captured much of the relevant pre-existing variability. These results do not eliminate bias, but they strengthen the inference that CIBI-related gains were not simply a reflection of higher general mathematics achievement.

### Implications for practice

Three implications follow. First, reasoning becomes more teachable when it is tied to visible student products and shared evaluative criteria; requiring documentation of claims, evidence, and warrants helps shift justification from an add-on to a norm (Alderton and Pratt, 2025). Second, the strong gains in D2/D3 suggest that high-leverage design work lies in institutionalizing evidence production and critique—not merely arranging students into groups—through prompts that press for “how do we know?” and “what would disconfirm this?” (Ellis et al., 2019; Zhuang and Conner, 2022). Third, the increasing fidelity trajectory suggests that modest professional learning paired with a stable lesson template may be sufficient to initiate productive routines, supporting feasibility under typical school constraints (Fry et al., 2025). This extends previous research by showing that structure can support reasoning without scripting student thinking.

### Limitations and future directions

The study’s scope invites cautious generalization. With one teacher per condition and one school, teacher effects and contextual factors cannot be fully separated from the model; replication across multiple teachers and schools—ideally with cluster-level assignment—would strengthen external validity. The five-week duration and absence of delayed posttests leave open questions about maintenance and transfer beyond the intervention window. The very high parallel-form association and internal consistency support form equivalence, yet may also indicate narrow task sampling; future versions should broaden item contexts to better represent transfer. Finally, richer process data (e.g., discourse or work-product trajectories) would allow direct tests of the hypothesized mechanism linking step enactment to gains in evidence and evaluation, aligning with calls to connect instructional design, enactment, and learning outcomes more tightly (Stylianides et al., 2024) and with psychological perspectives that emphasize the centrality of discourse and argumentation in learning interactions (Bova et al., 2024).

## Conclusion

Taken together, outcome and implementation evidence indicates that a structured, repeatable collaborative inquiry cycle can enhance elementary students’ mathematical reasoning, with particularly strong benefits for evidence use and evaluative justification and with larger gains among students who begin with weaker reasoning performance. More importantly, the study contributes to existing literature by clarifying stages often conflated in inquiry-based teaching, especially the distinction between generative exploration and justificatory/evaluative collaborative reasoning. Practically, the findings suggest that teachers need not only rich tasks but also explicit routines, prompts, and shared products that support claim-making, comparison, and validation. Theoretically, the study suggests that elementary learners may benefit from temporally organized reasoning actions rather than from undifferentiated exploration alone.

## Data Availability

The raw data supporting the conclusions of this article will be made available by the authors, without undue reservation.
