# Supplementary material for: A structured five-step collaborative inquiry approach to mathematical reasoning in elementary school: effects and implementation evidence
Source: Front Psychol. 2026 Aug 14;17:1813634. doi: 10.3389/fpsyg.2026.1813634 (PMC13521811; doi:10.3389/fpsyg.2026.1813634)
Supplement: Supplementary file 1 [file Supplementary_file_1.DOCX]

**Appendix A. CIBI Fidelity Observation Rubric (0–3 anchored ratings)**

Rating anchors: 0 = Not observed; 1 = Minimal/partial; 2 = Adequate; 3 = Strong/high-quality enactment. Overall fidelity is the mean of all 10 indicators.

| **CIBI step** | **Indicator** | **Score**  **(0–3)** | **Observer evidence notes**  **(brief)** |
| --- | --- | --- | --- |
| Step 1: Frame the Problem | 1. Claim & conditions made explicit (goal/unknowns/constraints surfaced). |  |  |
| Step 1: Frame the Problem | 2. “Explain why” norm established (students prompted to justify, not only answer). |  |  |
| Step 2: Conjecture & Plan | 3. Multiple conjectures/strategies voiced; group selects a focus claim. |  |  |
| Step 2: Conjecture & Plan | 4. Plan documented (cases/representations to test; criteria for ‘convincing’). |  |  |
| Step 3: Explore & Check Evidence | 5. Systematic testing with cases; counterchecks/counterexamples sought. |  |  |
| Step 3: Explore & Check Evidence | 6. Representations used as evidence (tables/diagrams/number sentences linked). |  |  |
| Step 4: Argue & Co-evaluate | 7. CER argument constructed (claim–evidence–reasoning/warrant articulated). |  |  |
| Step 4: Argue & Co-evaluate | 8. Peer critique and revision occur (questions/challenges lead to refinement). |  |  |
| Step 5: Reflect & Transfer | 9. Reflection summarizes what holds and why; reasoning practice named. |  |  |
| Step 5: Reflect & Transfer | 10. Near-transfer attempt completed (strategy applied to structurally similar task). |  |  |

**Appendix B. MRT Blueprint (Parallel Forms A and B)**

Each form contains 10 open-ended items (40 minutes; total score 40). All items require written reasoning.

| **Dimension** | **Operational indicator** | **Items per form** | **Points per item** |
| --- | --- | --- | --- |
| D1. Formulate claims/conjectures | State a claim/conjecture; specify givens/conditions; frame what needs to be shown | 2 | 4 |
| D2. Generate evidence & representations | Use cases/representations to test or support a claim; connect representations to quantities/relations | 3 | 4 |
| D3. Justify & evaluate | Provide warrants; critique arguments; use counterexample/boundary checks when appropriate | 3 | 4 |
| D4. Generalize/transfer | Abstract a strategy or structure; apply to a near-transfer task; articulate what remains invariant | 2 | 4 |

**Appendix C. Mathematical Reasoning Test (MRT) – Form A (Pretest) and Form B (Posttest)**

Administration: 40 minutes. Instructions: Show your thinking. Write words, draw diagrams, use tables, or write number sentences to explain why your answer makes sense.

**Form A (Pretest)**

**A1 (D1). 图形规律与猜想**

中文题目： 观察下列规律：第1个图有 3 个点，第2个图有 6 个点，第3个图有 10 个点，第4个图有 15 个点……
(1) 写出你对第n个图点数的猜想（用自己的话或用式子）。
(2) 解释为什么你的猜想合理（可以画图、列表或举例说明）。

English translation: Pattern conjecture: 3, 6, 10, 15, … Conjecture a rule for the nth figure and justify using evidence.

**A2 (D1). 条件与结论**

中文题目： 有两个不同的两位数，它们的和是 100。
(1) 你能提出一个“总成立”的结论吗？（例如：关于它们个位数、十位数或奇偶性等）
(2) 写出你的理由，并说明结论成立需要哪些条件。

English translation: Two different two-digit numbers sum to 100. State an always-true conclusion and justify with conditions.

**A3 (D2). 多位数乘法的算理**

中文题目： 小明算 24×15，用的方法是：24×15=24×(10+5)=24×10+24×5。
(1) 用你自己的话解释这种方法为什么对。
(2) 画一个示意图（如面积模型）或列出算式来支持你的解释。

English translation: Explain why 24×15 = 24×(10+5) = 24×10 + 24×5 using a diagram/equations.

**A4 (D2). 周长相同，面积比较**

中文题目： 两个长方形的周长都是 24 厘米。
长方形甲：长 7 cm，宽 5 cm；长方形乙：长 8 cm，宽 4 cm。
(1) 哪个面积更大？
(2) 不仅算出结果，还要解释“为什么会这样”（可以比较结构、画图或用表格）。

English translation: Two rectangles with perimeter 24 cm (7×5; 8×4). Compare areas and explain why.

**A5 (D2). 分数比较的证据**

中文题目： 比较 3/4 和 5/8 的大小。
(1) 写出你的判断。
(2) 用至少两种方法说明为什么（如画图、通分、数轴、等值分数等）。

English translation: Compare 3/4 and 5/8; justify using at least two methods.

**A6 (D3). 总是/有时/从不**

中文题目： 判断下面说法是“总是正确”“有时正确”还是“从不正确”，并给出理由：
“一个数先乘10再除以5，结果一定是原来的2倍。”

English translation: Always/sometimes/never statement; decide and justify.

**A7 (D3). 反例与修正**

中文题目： 有人说：“两个奇数相加，和一定是奇数。”
(1) 这个说法对吗？
(2) 如果不对，请给出反例，并解释为什么；如果对，请给出证明。

English translation: Use counterexample or proof to evaluate the claim.

**A8 (D3). 论证评价**

中文题目： 小红比较 2/3 和 3/5，她这样说：“2/3≈0.6多一点，3/5=0.6，所以 2/3>3/5。”
(1) 你同意吗？
(2) 请指出她的理由中哪些部分是证据，哪些部分需要更严谨，并给出一个更严谨的论证。

English translation: Critique and improve an argument comparing 2/3 and 3/5.

**A9 (D4). 迁移：同结构新题**

中文题目： 利用你在A3中解释的“分配律”思路，计算并解释：36×12=36×(10+2)=?
要求：写出算式并解释每一步为什么成立。

English translation: Transfer distributive reasoning to compute and explain 36×12.

**A10 (D4). 概括策略**

中文题目： 在做分数比较（如A5）时，你认为“什么时候通分更方便？什么时候用画图/数轴更方便？”
请用一个你自己编的例子说明你的判断，并解释理由。

English translation: Generalize when to use common denominators vs. diagrams/number lines; give an example and justify.

**Form B (Posttest)**

**B1 (D1). 图形规律与猜想**

中文题目： 观察下列规律：第1个图有 4 个点，第2个图有 7 个点，第3个图有 11 个点，第4个图有 16 个点……
(1) 写出你对第n个图点数的猜想。
(2) 解释为什么你的猜想合理（可以画图、列表或举例）。

English translation: Pattern conjecture: 4, 7, 11, 16, … Conjecture a rule for the nth figure and justify.

**B2 (D1). 条件与结论**

中文题目： 有两个不同的两位数，它们的差是 25（大数减小数）。
(1) 你能提出一个“总成立”的结论吗？
(2) 写出你的理由，并说明结论成立需要哪些条件。

English translation: Two different two-digit numbers differ by 25. State an always-true conclusion and justify.

**B3 (D2). 多位数乘法的算理**

中文题目： 解释为什么 18×23 = 18×(20+3) = 18×20 + 18×3。
要求：用文字+算式或面积模型说明。

English translation: Explain why 18×23 = 18×(20+3) = 18×20 + 18×3.

**B4 (D2). 周长相同，面积比较**

中文题目： 两个长方形的周长都是 30 厘米。
长方形甲：长 9 cm，宽 6 cm；长方形乙：长 10 cm，宽 5 cm。
(1) 哪个面积更大？
(2) 解释“为什么会这样”。

English translation: Two rectangles with perimeter 30 cm (9×6; 10×5). Compare areas and explain why.

**B5 (D2). 分数比较的证据**

中文题目： 比较 5/6 和 7/9 的大小。
(1) 写出你的判断。
(2) 用至少两种方法说明为什么。

English translation: Compare 5/6 and 7/9; justify using at least two methods.

**B6 (D3). 总是/有时/从不**

中文题目： 判断下面说法是“总是正确”“有时正确”还是“从不正确”，并给出理由：
“一个数先除以2再乘以6，结果一定是原来的3倍。”

English translation: Always/sometimes/never statement; decide and justify.

**B7 (D3). 反例与修正**

中文题目： 有人说：“两个偶数相加，和一定是偶数。”
(1) 这个说法对吗？
(2) 请给出证明或反例（并解释）。

English translation: Prove or refute (with explanation).

**B8 (D3). 论证评价**

中文题目： 小华比较 3/4 和 4/7，他说：“3/4 的分子和分母都比 4/7 大，所以 3/4>4/7。”
(1) 你同意吗？
(2) 请指出这段话的问题，并给出一个正确的论证。

English translation: Critique an incorrect argument about fractions and provide a correct justification.

**B9 (D4). 迁移：同结构新题**

中文题目： 利用你在B3中解释的思路，计算并解释：45×14=45×(10+4)=?
要求：写出算式并解释每一步为什么成立。

English translation: Transfer distributive reasoning to compute and explain 45×14.

**B10 (D4). 概括策略**

中文题目： 在判断“总是/有时/从不”题（如B6）时，你认为最有效的方法是什么？
请用一个你自己编的例子说明，并解释为什么这样能判断。

English translation: Generalize a strategy for always/sometimes/never statements; give an example and justify.

**Appendix D. MRT Scoring Rubric (0–4 per item)**

Each item is scored 0–4. Total score per form = sum across 10 items (0–40).

| **Score** | **Descriptor** |
| --- | --- |
| 4 | Mathematically valid claim/answer; reasoning is explicit and coherent. Evidence is appropriate and clearly connected to the claim via warrants. Computations support, not replace, reasoning. |
| 3 | Claim/answer is mathematically valid (or mostly valid) and reasoning is present but incomplete. Evidence is provided but warrants are partially implicit; a key condition/step may be missing. |
| 2 | Partial reasoning: correct computations or examples may appear, but the explanation is superficial or not clearly linked to the claim. Important warrants/conditions are missing. |
| 1 | Minimal attempt: isolated calculations or statements with little interpretable reasoning; major misconceptions or irrelevant work. |
| 0 | Blank or not interpretable as mathematical reasoning. |

**Appendix E. Parallel-Forms Equivalence Study (Class C) – Protocol**

Participants: Grade 4 Class C (n = 45), not involved in the intervention.

Design: Counterbalanced crossover. Students are randomly assigned to order AB or BA. Week 1: first form (40 min). Week 2: second form (40 min). No feedback is provided between administrations.

Analyses: (a) mean difference between forms with 95% CI; (b) Pearson correlation between total scores; (c) internal consistency (Cronbach’s alpha) for each form; (d) rater reliability (ICC) based on double-scoring a subset.

Criteria for equivalence: |mean difference| ≤ 1.0 point and r ≥ .80.

Observed results (pilot): Form A M = 26.82, SD = 7.85, α = .964; Form B M = 26.87, SD = 8.05, α = .967. The paired mean difference (A − B) was Δ = −0.04, 95% CI [−0.34, 0.25], t(44) = −0.31, p = .761, and total scores were strongly correlated (r = .993). No order effect was detected (AB vs. BA on A − B difference: t = 0.01, p = .995). Rater reliability for double-scored scripts was excellent (ICC(2,k) = .994).
